# Through service providers’ eyes: health systems factors affecting implementation of tuberculosis control in Enugu State, South-Eastern Nigeria

**DOI:** 10.1186/s12879-020-4944-9

**Published:** 2020-03-06

**Authors:** Daniel Chukwuemeka Ogbuabor

**Affiliations:** 1 Department of Health Administration and Management, University of Nigeria Enugu Campus, Enugu, Enugu State Nigeria; 2 Department of Health Systems and Policy, Sustainable Impact Resource Agency, Enugu, Enugu State Nigeria

**Keywords:** Tuberculosis, Control programme, Health systems, Constraints, Service providers, Nigeria

## Abstract

**Background:**

Well-functioning health systems are essential to achieving global and national tuberculosis (TB) control targets. This study examined health system factors affecting implementation of TB control programme from the perspectives of service providers.

**Methods:**

The study was conducted in Enugu State, South-eastern Nigeria using qualitative, cross-sectional design involving 23 TB service providers (13 district TB supervisors and 10 facility TB focal persons). Data were collected through in-depth, semi-structured interviews using a health system dynamic framework and analysed thematically.

**Results:**

Stewardship from National TB Control Programme (NTP) improved governance of TB control, but stewardship from local government was weak. Government spending on TB control was inadequate, whereas donors fund TB control. Poor human resources management practices hindered TB service delivery. TB service providers have poor capacity for data management because changes in recording and reporting tools were not matched with training of service providers. Drugs and other supplies to TB treatment centres were interrupted despite the use of a logistics agency. Poor integration of TB into general health services, weak laboratory capacity, withdrawal of subsidies to community volunteers and patent medicine vendors, poorly funded patient tracking systems, and ineffectual TB/HIV collaboration resulted in weak organisation of TB service delivery.

**Conclusion:**

Health systems strengthening for TB control service must focus on effective oversight from NTP and local health system; predictable domestic resource mobilisation through budgets and social health insurance; training and incentives to attract and retain TB service providers; effective supply and TB drug management; and improvements in organization of service delivery.

## Background

Well-functioning health systems are essential to achieving global and national tuberculosis (TB) control targets [1]. The global targets are 90% reduction in the number of TB deaths and 80% reduction in TB incidence rate (number of new TB cases per 100, 000 population per year) relative to 2015 levels [1]. In 2018, about 10 million people fell ill with TB, resulting in 1.5 million deaths [1]. The reduction in TB incidence rate between 2015 and 2018 was only 6.3%, significantly lower than the End TB Strategy milestone of 20% between 2015 and 2020 [1]. The End TB Strategy identified integrated, patient-centred care, bold policies and supportive systems as pillars to end TB by 2030 [2]. Comprehensive changes to policies and regulation, organisational structures, processes and relationships and effective use of resources are needed to achieve and sustain goals of TB control programme [3, 4]. Nonetheless, low- and middle-income countries (LMICs) still struggle with health system strengthening for TB control.

Health systems factors that strengthened TB control include effective national strategies [5–7]; strong stewardship from district government [8]; donor funding [5, 8–13]; inclusion of TB in social health insurance schemes [14, 15]; earmarked funding for TB [16]; medical vouchers and subsidies [17, 18]; providers’ satisfaction with directly observed treatment short course [19]; incentives to programme staff [20]; patient-centred approaches and intersectoral collaboration [6, 16, 19]; motivated and dedicated healthcare workers [16, 19]; effective supply and drug management system [8, 21]; use of dedicated logistics agency to distribute TB drugs [9, 22]; and electronic reminders to improve treatment adherence [17].

Constraints to TB control include perception that TB control is less important than other public health programs [20, 23]; weak accountability relationship between provincial/regional and district TB programme management [7, 12, 24]; insufficient community involvement [25]; and low public spending on TB [5–7, 9–13]. TB control have been limited by lack of skilled staff [4, 10, 11, 19, 22]; lack of incentives for service providers [4, 5, 10]; lack of utilization of various levels of health staff and health facilities [25]; poor attitude and weak commitment of health workers towards deployment to TB services [22, 23], inadequate training [10, 11, 19], and lack of laboratory staff [5, 22, 23]. Poor TB data management capacity [22]; frequent revisions of reporting formats [5]; and ineffective electronic recording and reporting system [14]. Shortages of TB drug [7, 22] and unavailability of laboratory supplies and equipment hinder TB service delivery [7, 11, 22]. Poor adherence to GeneXpert algorithm, interrupted supply of cartridges, lack of replacement of damaged modules, poor maintenance, and poor recording and reporting limit usefulness of GeneXpert in TB diagnosis [14, 26, 27]. Poor adherence to national guidelines [11, 22, 23]; dilapidated infrastructure [5, 12, 23] weak TB/HIV integration [10, 12, 14, 24, 28]; lack of public-private mix [10, 12, 23] and poor integration into general health services hinder effective TB service delivery [5, 29, 30].

Nigeria’s national strategic plans for TB control emphasised health system strengthening [31]. However, after over two decades of TB control, Nigeria has about 60% funding gap and 24% TB case notification rate, and is one of ten countries accounting for 80% gap in TB notification globally [1]. Nigeria remains a high TB burden country with incidence rate of 219 per 100,000 population [1]. In 2018, about 429,000 people fell ill with TB, resulting in 157,000 deaths; domestic resource mobilisation for TB was considerably low at 8%; and about 71% of TB patients faced catastrophic costs in Nigeria [1]. The southeast region of Nigeria was particularly problematic with median TB case notification rate of 16% (range, 10 to 18%) [32]. In Enugu State, TB case notification rate was also 16% [32]. Yet, studies using systems thinking to analyse implementation of TB control in Nigeria are scarce. The study therefore examined health system factors affecting implementation of TB control programme in Enugu State, Nigeria from the perspectives of district-level service providers. Such evidence would inform policy changes to ensure universal access to TB services in low resource, high TB burden settings.

## Methods

### Conceptual framework

**Fig. 1:**
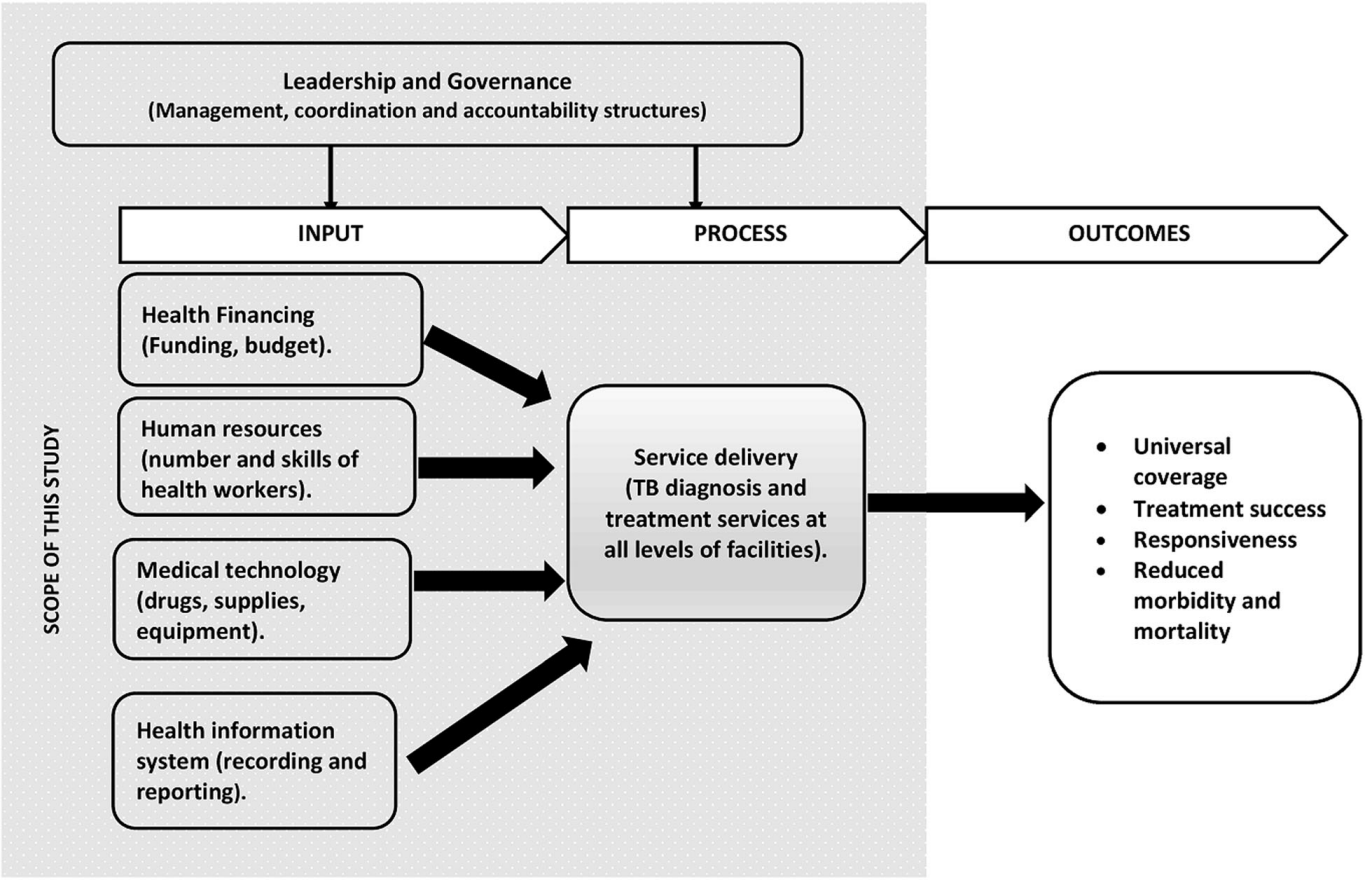
Health systems dynamic framework for tuberculosis control

The study was guided by health systems framework by Van Olmen and colleagues [33]. The framework builds on World Health Organization’s six building blocks of a health system [34] and uses input, process, outcomes and goals to explain health systems dynamics (Fig. 1). The goals of TB control are reductions in TB mortality and incidence. Universal coverage, treatment success and responsiveness are outcomes of optimal TB control programme. TB diagnosis and treatment is considered service delivery block and equals the process in the framework. Health financing, human resources, medical technology and health management information system (HMIS) serve as inputs into service delivery. Governance, which entails policy guidance, policy coordination, regulation of different functions, optimal allocation of resources and accountability, influences both the input and process. This framework was chosen because it explains how interactions of governance, inputs and process would lead to improved implementation of TB control and better health outcomes.

### Study setting

The study was conducted in Enugu State, South-east Nigeria. Enugu State is one of five geo-demographically and culturally homogenous states in southeast region of Nigeria. Enugu State was purposively chosen for this study because its 2018 TB case notification rate equals the median regional case TB notification rate of 16% [32]. The state consists of 17 local government areas (LGAs); 12 of which are predominantly rural areas. The National TB Control Programme (NTP) is headed by a state control officer. Each local government area (LGA) has a TB supervisor (LGTBS). The state has 71 and 41 TB treatment centres in public and private health facilities respectively. Publicly owned TB treatment centres comprise primary, secondary and tertiary health facilities. Each treatment centre has a TB focal person.

### Research design

The study adopted a cross-sectional, qualitative study design using in-depth, semi-structured interviews.

### Study population and sampling strategy

Study subjects were LGTBS and facility TB focal persons in Enugu state. All 17 LGTBS were included in the study. However, data from LGTBSs was saturated after the thirteenth interview [35]. To select facility TB focal persons, the 17 LGAs were categorized into low (8 LGAs) and high (9 LGAs) case finding districts based on case notification, an important indicator of successful TB control programme [1, 31]. From each category, 5 LGAs were purposively selected. One TB treatment centre was purposively selected from each LGA. The TB focal persons in the selected treatment centres were interviewed. Maximum variation sampling was used to ensure that both rural and urban LGAs and all levels of facilities were represented (Table 1).

**Table 1:** Details of study participants

Participants	District	Number of participants	Number of participants by level of care/health facility			
			***Primary health centre***	***Secondary health centre***	***Tertiary health centre***	***Faith-based hospital***
LGTBS	Urban	4	4			
	Rural	9	9			
Facility focal persons	Urban	4	2	1	1	1
	Rural	6	4	1		
Total		23	19	2	1	1

### Data collection

Twenty-three participants were interviewed between January and April 2017 using pre-tested, semi-structured in-depth interview guide (Additional file 1). The interview questions were informed by the conceptual framework, adapted to TB control programme. Participants were identified using health officials as gatekeepers. Interviews were held in offices or health facilities, conducted in English and lasted about 60 min. All interviews were audiotaped, transcribed verbatim and verified by participants for accuracy of transcription.

### Data analysis

Data were analysed using a framework approach. Deductive and inductive strategies were used to code the transcripts in NVivo 11 software. Main themes were deduced from health systems’ building blocks. Inductive codes were generated by familiarization with data and assigning codes to emerging themes. Coding was carried out by two persons and inconsistencies resolved by consensus. The LGTBS validated the study findings during a programme review meeting, which improved the validity of the findings.

### Ethical consideration

The study was approved by Health Research Ethics Committee of Enugu State University Teaching Hospital, Parklane, Enugu, Nigeria. Written, informed consent was obtained from all participants.

## Results

Findings are presented thematically using health system building blocks deduced from the study’s conceptual framework (Table 2).

**Table 2:** Summary of key health systems enablers and constraints to implementation of TB control in Enugu State, South-eastern Nigeria

Key enablers	Key constraints
***Leadership and governance***	
Stewardship from state-level NTP	Weak bureaucratic accountability from local health system.
Programme management support	Low government attention to TB control program
Regular performance review and coordination	
***Health financing***	
External funding for TB control from donors	Budgeted funds are not released to TB control program at the state level.
	Absence of TB in local governments’ budget.
	TB supervisors’ motorbikes are not replaced for several years.
***Human resources***	
Supportive supervision of facility TB focal persons.	Unwillingness of health workers to work in TB control programme
	Frequent re-deployment of skilled TB service providers
	High number of untrained TB service providers.
	Health workers are owed several months of salaries
***Health technology***	
Use of dedicated logistics agency for drug distribution.	Logistics agency dumped drugs meant for entire local government in one location.
	Drug kits does not meet needs of extrapulmonary TB patients and those weighing more than 70 kg.
	Shortage of human immunodeficiency virus (HIV) test kits.
***Health information system***	
Availability of recording and reporting tools	Change in tools are not matched with training of service providers.
Adaptation of tools to strategies in TB control.	
Introduction of electronic recording and reporting system	
***Service delivery***	
Availability of functional microscopic centre	Stigma by health workers
Introduction of GeneXpert	Concern for contracting TB
Engagement of community volunteers and patent medicine vendors	Lack of incentives to attract health workers
Reduction in duration of treatment from 8 to 6 months	Many TB treatment centres lack of TB laboratory
	Poorly functioning GeneXpert.
	Weak patient tracking system
	Withdrawal of incentive for community volunteers and patent medicine vendors.
	Limited number of TB/HIV collaborative sites.

### Leadership and governance

Stewardship from the State and Local Governments emerged as key theme. All LGTBS received regular managerial oversight from the state TB programme. Similarly, most facility TB focal persons stated that *“TB supervisors are always available when we need their advice, or we have questions or clarifications”* (FP5). Quarterly review meetings offered opportunity for joint programme planning and coordination and holding LGTBS accountable. LGTBS serve under Primary Health Care Coordinators (PHCCs), who manage LG’s health system. Nonetheless, most PHCCs, except those who were LGTBS prior to their appointment as PHCC, were unsupportive of TB control. It was explained that “*because money does not come from the state … , TB control program is not important*” (TBS3) to local government officials. Furthermore, facility TB focal persons noted that PHCC rarely visited health facilities.

### Financing of TB control program

Most participants stated that NTP has been donor-driven while public spending on TB control is low. As one supervisor stated, *“TB programme is donor-driven. If I should say my mind, the government is not doing what it should”* (TBS1). At the state level, allocated funds are not released to TB programme. At LG level, unlike other public health programs, TB control programme does not have a budget. Government officials feel that *“we are collecting dollars from these development partners; why come to look for naira”* (TBS1); and *“we don’t have anything (incentive) coming out of the program”* (TBS10). Although LGs agreed to replace TB supervisors’ motorcycles, no LG ever supplied motorcycle to LGTBS, which limits supervision, community mobilization, and contact and defaulter tracing. One facility TB focal persons lamented *“I have written the local government chairman severally through the head of department of health, for financial support to build TB laboratory. They both signed, but till now, I have not seen anything from them”* (FP3).

### Human resources for health

Inadequate staffing, posting and transfer, supervision, remuneration and training emerged as key themes. Inadequate staffing stemmed from unwillingness of health workers to work in NTP due to stigma and lack of incentives. Consequently, TB service providers reported high workload: *“my workload is high. It is not only TB services that I provide. I am also the anti-retroviral therapy focal person”* (FP7). Most participants observed that trained TB service providers are frequently transferred out of TB treatment centres. Few participants noted the social restriction to the posting or transfer of female health workers. Most participants agreed that supervision was supportive: *“if there are things we are doing wrong, they correct us, … and if there are things we are doing very well, they still commend us”* (FP5). However, supervision is limited by *“lack of functional motorbike and allowances to cover fuel and maintenance”* (TBS5). Most LGAs were owing staff several months’ salaries. Yet, financial incentives are scare especially among facility TB focal persons who *“do not get any incentive”* (TBS3). Most participants noted that there are many untrained TB focal persons. External training was preferred to on-the-job training because *“they do not give us anything, except when we go for seminar”* (FP4).

### Health information system

Most participants indicated that recording and reporting tools were available but frequently changed without prior training of service providers. One participant stated: *“they used to change the pattern and the format, which we find very difficult to cope with”* (FP3). Modifications in tools were adaption to changes in TB control strategies: *“we changed from eight months to six months treatment. Now we are addressing HIV, drug resistant TB and presumptive cases”* (TBS5). Poor recording and reporting also resulted from work overload and lack of incentives. Electronic Tuberculosis manager (ETBM) has been rolled out to high burden facilities and LGTBS. Most participants perceived that ETBM would enable timely and complete recording and reporting. Few participants anticipated that ETBM would add to workload of service providers.

### Medical / drug supplies

Most participants stated that TB drug supplies were interrupted during the preceding year: *“a quarter would pass without supply”* (FP6). Although, a logistics organization was contracted by the NTP to supply drugs directly to TB treatment centres, *“they cut corners by dropping all the supplies meant for facilities in an LGA at one facility and expect individual facilities to come and pick their supplies there.”* (TBS1). Most TB treatment centres coped by borrowing drugs from other health facilities. Few participants explained extrapulmonary TB patients and TB patients weighing above 70 kg required more than one drug kit to meet their dosage requirements. A facility TB focal person remarked that *“we have to de-kit in order to make it up for six months”* (FP6). Human immunodeficiency virus (HIV) test kits were no longer supplied to TB treatment centres. A LGTBS asserted: “*for the past 2 years, we have not received any HIV test kits*” (TBLS3). Consequently, some health workers procure HIV test kits and charge fees for HIV screening.

### Service delivery

Most providers stated that TB is poorly integrated into general health services due to stigma by health workers, concern for contracting TB and lack of financial incentives. As explained, *“some health workers refuse participating in TB control because of fear that they might contract TB”* (TBS5); or *“that nothing is coming out of it (TB programme), unlike malaria, monitoring and evaluation, and immunization”* (TBS9). Most providers reported availability of functional microscopic centres. However, some providers indicated that TB diagnosis is constrained by lack of space, damaged microscope and lack of laboratory staff: *“I have up to 10, but only 1 is functional”* (TBLS2). Lack of dispatch riders, breakdown of machine, varying days of operations, and absence of support for sputum transport constrained use of GeneXpert for TB diagnosis. Withdrawal of incentives constrained involvement of community volunteers and patent medicine vendors. As one participant stated, *“Usually, community volunteers received monthly stipends, but since GHAIN (a development partner) stopped it and government could not sustain it, all of them declined”* (FP1). Also, *“only very few of patent medicine vendors, who are health workers, still refer suspects to TB programme”* (TBLS6). Some providers revealed that patient tracking systems is weak due to poor funding for contact and defaulter tracing, while TB/HIV collaboration was limited to comprehensive sites.

## Discussion

Whereas stewardship from the NTP improved district TB service delivery, stewardship from local government (LG) was weak despite existence of memorandum of understanding between LGs and NTP. Effective bureaucratic relationship between state and LG TB programmes in this study contradicts evidence of poor stewardship in India, Ghana and South Africa [7, 12, 24]. In our setting, accountability relationship was enhanced by programme management support, supportive supervision and quarterly performance review meetings. Nevertheless, the weak stewardship from the LG confirms previous evidence that TB control program is perceived as less important than other public health programmes [20, 23] but contrasts evidence of strong political commitment from district governments in Pakistan [8]. Unmet expectation of local officials of financial incentives from NTP and perception that partners support TB control explained low responsiveness of LGs. Since governance affects input and process of TB control, LG officials need to strengthen stewardship of and become more responsive to TB control program.

This study also found that government spending on TB control was inadequate, which is similar to evidence from several studies where donors, instead of governments, drive TB control efforts [5–13]. In this study, non-release of budgeted funds at the state-level and complete absence of TB in LG budgets resulted in persisting TB funding gap and unlikelihood of sustainability of TB control. As external funding declines, all tiers of government in Nigeria need to improve financing of TB control through budgets. Also, Nigeria’s efforts to decentralise social health insurance to states presents an opportunity to include TB in social health insurance schemes [14, 15], given that Nigerian TB patients incur catastrophic costs [36, 37]. Moreover, it might be helpful to consider medical vouchers and subsidies for poor TB patients [17, 18].

This study’s findings revealed that poor human resources management practices and inadequate training of TB service providers hindered TB control. These findings are consistent with lack of skilled staff [4, 10, 11, 22]; lack of incentives for TB service providers [5, 10]; inadequate training [10, 11, 19]; and poor attitude and weak commitment of health workers towards deployment to TB services [22, 23]. In contrast, evidence from European countries show that skilled and motivated healthcare workers enabled TB control [16]. Strategies that aim at improving TB control workforce must address shortages of staff, stigma by health workers and lack of incentives to TB service providers. Additionally, broad contextual factors underlying human resource crisis especially social restriction to deployment of female health workers and delayed payment of staff salaries warrant attention of decision makers.

The findings revealed that TB service providers have poor capacity for recording and reporting TB data due to mismatch between changes in tools and training of service providers, which is similar to evidence from Ethiopia and Nigeria [5, 22]. Even though, changes in tools were adaptation to new developments in TB control program, lack of training meant that TB service providers did not completely fill tools and required constant supportive supervision. A strategy to improve data management was introduction of electronic recording and reporting system. However, experiences from South Africa suggest that operational challenges limited effectiveness of electronic recording and reporting system [14]. Nigeria’s NTP would need to improve on-the-job capacity building of service providers on data management, while addressing the limitations of electronic recording and reporting system.

The findings of this study that drugs and other supplies to TB treatment centres were interrupted are consistent with evidence of shortages of TB drug and laboratory supplies elsewhere [7, 11, 22], but contrast evidence of effective supply and drug management in Pakistan [8]. Although use of dedicated logistics agency to distribute TB drugs improved TB drug supply system [9, 22], experiences in our study area reveal that TB drugs for an entire district were often dumped in one health facility. Equally, absence of drug kits tailored to extrapulmonary TB patients and TB patients weighing 70 kg and above. Hence, service providers must augment one drug kit to meet their dosage requirements, which depletes the stock. Sustainable TB drug supply system would entail strengthening the logistics agency and improving the capacity of NTP to forecast TB drug needs.

Weak TB service delivery system was found as a key constraint to TB control. Consistent with findings of previous studies [5, 29, 30], integration of TB into general health services was weak. Social stigma and concern for contracting TB meant that health workers refuse deployment to and participation in TB control programmes. Also, the findings of weak laboratory capacity due to lack of space, damaged microscopes, attrition of laboratory staff and operational challenges of GeneXpert, are like evidence from previous studies [5, 11, 14, 22, 23, 26, 27]. Equally, poor involvement of community volunteers and patent medicine vendors due to withdrawal of financial incentives hindered TB service delivery. Although TB service providers have used electronic reminders to improve treatment adherence similar to China’s experience [17], weak patient tracking systems resulted from poor funding and lack of transportation. Notwithstanding existence of guidelines for TB/HIV integration, weak TB/HIV collaboration is similar to evidence from other studies [10, 12, 14, 24, 28]. Policies to improve TB service delivery system must address stigma among health workers, strengthen laboratory capacity, incentives to community volunteers and patent medicines vendors, improve funding of patient tracking and bolster TB/HIV integration.

This study adds to the growing scholarship using health systems lens to examine disease control programmes. Particularly, application of systems thinking provided useful analytical tool to explain how health system strengthening enables or constrains TB control in high TB burden, low-resource settings. However, as the participants were limited to frontline, district-level TB service providers in one sub-national context, the findings may not be generalizable to other settings. Nonetheless, this study does not aim to generalize but to provide evidence to inform policy changes to ensure universal access to TB services in low-resource settings facing similar approaches. Given the emphasis on people-centred health system, perceptions and experiences of consumers TB service delivery systems would be an area of future research.

## Conclusion

In conclusion, the findings of this study illustrate how health system factors drive control of TB in low resource settings. Given that governance influences both inputs and service delivery, governing TB control service delivery would entail effective oversight from NTP and local health system. Sustainable financing of TB would depend on stable and predictable domestic resource mobilisation through budgets and social health insurance. To attract and retain staff, human resource strategy must address training needs, lack of incentives to, and stigmatization of TB service providers. Furthermore, it is vital to strengthen TB drug and commodities’ supply system and organization of service delivery.

## Supplementary information

**Additional file 1.** Interview guide.

## Data Availability

The dataset supporting the conclusions of this article is included within the article.

## Abbreviations

ETBM: Electronic Tuberculosis Manager
FP: Focal person
HIV: Human immunodeficiency virus
LG: Local government
LGA: Local government area
LGTBS: Local government tuberculosis supervisor
LMICs: Low- and middle-income countries
NSP-TB: National strategic plan for tuberculosis control
NTP: National TB control programme
PHCC: Primary health care coordinator
TB: Tuberculosis
TB/HIV: Tuberculosis/ Human immunodeficiency virus
UHC: Universal health coverage
WHO: World Health Organisation
