# Supplementary material for: Through service providers’ eyes: health systems factors affecting implementation of tuberculosis control in Enugu State, South-Eastern Nigeria
Source: BMC Infect Dis. 2020 Mar 6;20:206. doi: 10.1186/s12879-020-4944-9 (PMC7060534; doi:10.1186/s12879-020-4944-9)
Supplement: Supplementary file 1 — Additional file 1. Interview guide. [file 12879_2020_4944_MOESM1_ESM.docx]

**Interview guide**

1. What is your role in TB control programme?
2. How does the national TB control programme (NTP) support or hinder your role in TB control?
   1. Probe for support received from NTP at national, state or local government (LG) level
3. Describe how your Local Government participate in TB control.
   1. Probe for the role of chairman of LG
   2. Role of primary health care (PHC) coordinator
4. How is TB control programme funded in your LG funded?
   1. Probe for state budget support
   2. Probe for LG budget support
5. How are TB services delivered in your facility/LG?
   1. Probe for role of different health facilities
   2. Probe for availability of human resources, drugs, laboratory supplies, equipment, recording and reporting tools, monitoring and supervision
   3. involvement of other providers
   4. TB/HIV collaboration
6. What kind of support do you think the facility focal persons need?
7. What challenges that the TB service providers face at the facility, local government or state levels?
